# The immune response against *Chlamydia suis* genital tract infection partially protects against re-infection

**DOI:** 10.1186/s13567-014-0095-6

**Published:** 2014-09-25

**Authors:** Evelien De Clercq, Bert Devriendt, Lizi Yin, Koen Chiers, Eric Cox, Daisy Vanrompay

**Affiliations:** Department of Molecular Biotechnology, Faculty of Bioscience Engineering, Ghent University, Coupure Links 653, B-9000 Ghent, Belgium; Laboratory of Immunology, Faculty of Veterinary Medicine, Ghent University, Salisburylaan 133, B-9820 Merelbeke, Belgium; Department of Pathology, Bacteriology and Poultry Diseases, Faculty of Veterinary Medicine, Ghent University, Salisburylaan 133, B-9820 Merelbeke, Belgium; College of Veterinary Medicine, Sichuan Agricultural University, Ya’an, 625014 China

## Abstract

The aim of the present study was to reveal the characteristic features of genital *Chlamydia suis* infection and re-infection in female pigs by studying the immune response, pathological changes, replication of chlamydial bacteria in the genital tract and excretion of viable bacteria. Pigs were intravaginally infected and re-infected with *C. suis* strain S45, the type strain of this species. We demonstrated that S45 is pathogenic for the female urogenital tract. *Chlamydia* replication occurred throughout the urogenital tract, causing inflammation and pathology. Furthermore, genital infection elicited both cellular and humoral immune responses. Compared to the primo-infection of pigs with *C. suis*, re-infection was characterized by less severe macroscopic lesions and less chlamydial elementary bodies and inclusions in the urogenital tract. This indicates the development of a certain level of protection following the initial infection. Protective immunity against re-infection coincided with higher *Chlamydia*-specific IgG and IgA antibody titers in sera and vaginal secretions, higher proliferative responses of peripheral blood mononuclear cells (PBMC), higher percentages of blood B lymphocytes, monocytes and CD8^+^ T cells and upregulated production of IFN-γ and IL-10 by PBMC.

**Electronic supplementary material:**

The online version of this article (doi:10.1186/s13567-014-0095-6) contains supplementary material, which is available to authorized users.

## Introduction

*Chlamydia suis* is widespread in commercial pig production and causes important economic losses [1]. *C. suis* infections are associated with a variety of diseases including conjunctivitis [2], rhinitis [3], pneumonia [3,4], enteritis [5,6], periparturient dysgalactiae syndrome (PDS) [7] and numerous reproductive disorders [1,7-10] such as abortion, mummification, perinatal and neonatal mortality, repeat breeding, vaginal discharge and endometritis in addition to orchitis, epididymitis and urethritis in boars [11]. *C. suis* has also been detected in the semen of boars, suggesting the possibility of venereal transmission during mating or artificial insemination [1,12,13]. Furthermore, *C. suis* has frequently been identified in asymptomatic carriers [14-16].

Experimental infection of gnotobiotic and conventionally raised pigs with *C. suis* proved its pathogenic potential for the porcine respiratory system [3,4,17], gastrointestinal tract [5,6,18] and eyes [2]. In contrast, the exact role of *C. suis* in reproductive problems still has to be defined. To our knowledge, experimental genital tract infection of pigs with *C. suis* has never been performed before. Nevertheless, considering the possibility of venereal transmission, genital *C. suis* infections and re-infections are probably common in breeder sows, since they are mated or inseminated a minimum of twice a year and generally two to three inseminations per oestrus are carried out. In the present study, conventionally raised *Chlamydia*-negative pigs were intravaginally infected and/or re-infected with *C. suis* strain S45, the type strain of this species. The aim of this study was to reveal the characteristic features of a genital *C. suis* infection and re-infection in female pigs by studying the immune response, pathological changes, replication of chlamydiae in the genital tract and excretion of viable bacteria.

## Materials and methods

### *Chlamydia suis*

*C. suis* strain S45 (kindly donated by A. Andersen, National Animal Disease Center, USDA Agriculture Research Service, Ames, Iowa) was used to infect the pigs. This strain was isolated in the late 1960s from feces of an asymptomatic Austrian pig [19]. The bacteria were grown in McCoy cells using standard techniques [20]. The 50% Tissue Culture Infective Dose (TCID_50_) was determined by the method of Spearman and Kaerber [21]. TCID_50_/mL is routinely used for titrating intracellular organisms like viruses in cell cultures and it correlates with IFU/mL as examined by Beeckman et al. [22].

### Infection experiment

Fifteen 9-week-old conventionally bred female pigs (Belgian Landrace) were randomly divided into three groups of five pigs. Antibodies against *Chlamydiaceae* were absent as determined by a *C. suis* ELISA using purified elementary bodies (EBs) of strain S45. Nasal, rectal and vaginal swabs did not contain chlamydial bacteria as determined by culture on McCoy cells. The animals were housed in isolation units and fed *ad libitum* with a commercial starting diet. At day 0, all pigs were anesthetized by intramuscular injection of Zoletil® 100 (Virbac Animal Health, Louvain La Neuve, Belgium) in 2% Xylazine-M® (VMD, Arendonk, Belgium). The control group and the infection group were inoculated with phosphate-buffered saline (PBS). The re-infection group was infected by intravaginal injection of 1 × 10^7^ TCID_50_ of *C. suis* strain S45. Intravaginal inoculation was performed by inserting an artificial insemination pipette connected to a syringe into the vagina and injecting the bacterial suspension in 1 mL PBS or injecting simply 1 mL PBS (controls). At day 56, pigs were anesthetized again, whereafter the control group was inoculated with PBS and the infection group and the re-infection group were infected intravaginally with *C. suis* strain S45 (1 × 10^7^ TCID_50_). All groups were euthanized at day 77 by intravenous injection of an overdose of pentobarbital (70 mg/kg; Nembutal®, Ceva Santé Animale, Maassluis, the Netherlands) followed by exsanguination.

Vaginal swabs in 2 mL *Chlamydia* transport medium for chlamydial isolation and in 2 mL PBS with protease inhibitor for mucosal antibody detection were collected weekly. Swabs were stored at −80 °C until analysis. Blood for antibody detection was sampled from the jugular vein (*v. jugularis*) weekly. Blood samples were stored overnight at room temperature and centrifuged (9300 × *g*, 20 °C, 10 min) for serum collection. Sera were stored at −20 °C until tested. At euthanasia, pigs were examined for gross lesions that were scored as none (0), slight (1), moderate (2) or severe (3). Samples of the spleen, liver, caecum, urethra, vagina, cervix, corpus uteri, uterine tubes, oviducts and ovaries were imbedded in methylcellulose medium, frozen in liquid nitrogen and stored at −80 °C until preparation of cryostat tissue sections for localization and quantification of the infection. Samples from the same tissues were taken for histopathology. The experimental procedure was approved by the animal care and ethical committee of Ghent University (EC2012/078).

### Chlamydial antigen detection

Vaginal swabs in *Chlamydia* transport medium were shaken for 1 h at 4 °C, centrifuged and cultured in McCoy cells grown on 13 mm cover slips in *Chlamydia* Trac Bottles (International Medical, Brussels, Belgium) as described by Vanrompay et al. [20]. Additionally, cells were washed twice with 1 mL phosphate buffer supplemented with 0.003% DEAE-dextran before the inoculum was added to the cells. Chlamydial growth was monitored using the IMAGEN™ direct immunofluorescence staining (Oxoid, Drongen, Belgium) at 6 days post inoculation. Cryostat tissue sections (5 μm) were also stained by use of the IMAGEN™ immunofluorescence test. All slides were examined by immunofluorescence microscopy (BX41 Olympus, 600×). Positive cells were counted in five microscopic fields. The calculated mean was presented as a score ranging from 0 to 6. Score 0 indicated a negative result. Score 1 and 2 indicated 1 to 5 and 6 to 10 elementary bodies (EBs) per microscopic field in the absence of inclusions, respectively. Score 3 represented more than 10 elementary bodies and 1 inclusion-positive cell per microscopic field. Score 4, 5 and 6 indicated 1 to 5, 6 to 10 and more than 10 inclusion-positive cells per microscopic field, respectively.

### Antibody responses

Sera were heat inactivated at 56 °C during 30 min and subsequently pretreated with kaolin to reduce background activity [23]. Vaginal swabs in PBS with protease inhibitor were shaken for 1 h at room temperature and centrifuged. Isotype-specific serum and mucosal antibody titers were determined in an ELISA using purified S45 EBs as antigen. Maxisorp 96-well microtiter plates were coated with purified *C. suis* strain S45 EBs (1 × 10^7^ TCID_50_ per well) diluted in PBS. Plates were blocked overnight (4 °C) with PBS supplemented with 5% bovine serum albumin (BSA). Serum and mucosal antibody titers were determined using twofold dilution series in dilution buffer (PBS + 3% BSA + 0.05% Tween®20), starting at a dilution of 1:15. Antibody isotype titers were determined using monoclonal antibodies against swine IgA (mAb 27.8.1), IgG (mAb 23.3.1b) and IgM (mAb 28.4.1) [24] at a dilution of 1:15, 1:20 and 1:50, respectively, followed by an 1:5000 dilution of biotinylated anti-mouse IgG (H + L) (Dako, Glostrup, Denmark). Subsequently, the plates were incubated with 1:2500 diluted peroxidase-labeled streptavidin (Zymed Laboratories, San Francisco, USA). Finally, the substrate and chromogene ABTS (2, 2′-Azino-di(3-ethylbenzthiazoline-6-sulfonate); KPL, Maryland, USA) was added. Antibody titers were defined as the reciprocal of the highest dilution that gave an absorbance reading at 405 nm above the cut-off value (mean absorbance of seronegative pig serum + twice the standard deviation). Positive and negative control sera and mucosal secretions originated from a former experimental infection in pigs [25].

### Proliferative responses of PBMC and MC

At 7 and 10 days post infection (dpi) or re-infection, peripheral blood mononuclear cells (PBMC) were isolated from heparinized blood samples collected from the jugular vein. Isolation of PBMC was performed by density gradient centrifugation (500 × *g*, 18 °C, 25 min) on Lymphoprep™ (Axis-Shield, Oslo, Norway). After lysis of erythrocytes in ammonium chloride, the cells were washed and resuspended in leukocyte medium (RPMI-1640 (Life Technologies, Merelbeke, Belgium) supplemented with 5% heat-inactivated fetal calf serum (Life Technologies), 5 × 10^−5^ M β-mercaptoethanol (Life Technologies), 1% non-essential amino acids (Life Technologies), 1% sodium pyruvate (Life Technologies), 1% L-glutamine (Life Technologies), 1% penicillin-streptomycin and 1% kanamycin). At euthanasia, monomorphonuclear cells (MC) were isolated from the spleen, the cervical lymph node (*cervicalis superficialis)* and the pelvic lymph nodes (*iliaci mediales*, *iliaci laterales, sacrales* and *anorectales*). Isolation of MC was performed by tearing the tissues apart, after the surrounding fat was removed. Subsequently, the erythrocytes were lysed with ammonium chloride. After centrifugation (270 × *g*, 4 °C, 10 min), the cells were washed and resuspended in leukocyte medium without β-mercaptoethanol. PBMC or MC were grown in 96-well tissue culture plates at a concentration of 5 × 10^5^ cells/well. Proliferation was tested by adding 10^5^ viable *C. suis* purified S45 EBs, 10 μg concanavalin A (ConA) (positive control) or medium (negative control) to the wells. Each condition was tested in duplicate. The cells were incubated at 37 °C in a humidified atmosphere with 5% CO_2_. ConA- or antigen-induced proliferation was measured by incorporation of 1 μCi/well of ^3^H-thymidine (Amersham ICN, Bucks, UK) during the last 16 h of culture, on day 3 and day 4, respectively. Cells were harvested onto glass fiber filter strips (Perkin Elmer, Life Science, Oosterhout, The Netherlands) with a cell harvester (Skatron, Liers, Norway). Radioactivity incorporated into the DNA was measured with a β-scintillation counter (Perkin Elmer). The stimulation index (SI) was defined as the ratio of the mean counts per minute of stimulated PBMC or MC versus the mean counts per minute of the negative control.

### Flow cytometric analysis

PBMC and MC from the spleen, cervical and pelvic lymph nodes were isolated as described above. Flow cytometry was used to analyze the immune cell (sub)populations in the blood at different time points post infection and of the spleen, cervical and pelvic lymph nodes at euthanasia. T cell populations (CD3^+^CD4^+^CD8^−^, CD3^+^CD4^−^CD8^+^, CD3^+^CD4^+^CD8^+^, CD3^+^CD4^−^CD8^−^, T cells with a γδ T cell receptor), B cells (MHCII^+^CD21^+^, MHCII^+^IgM^+^), monocytes (MHCII^+^SWC3^+^), NK cells (CD3^−^CD4^−^CD8^+^) and plasmacytoid dendritic cells (pDC) (CD3^−^CD4^+^CD8^−^) were quantified. However, for the lymphoid tissues the CD3^−^CD4^+^CD8^−^ population does not exist entirely of pDC, but also contains an unknown lineage, presumably of myeloid origin [26]. Based on the used markers, it was impossible to distinguish between both populations. PBMC and MC (10^6^ cells) were incubated for 20 min in staining buffer (RPMI-1640 + 1% heat-inactivated FCS) with optimal concentrations of mAbs (Table 1). Cells stained with isotype-matched irrelevant mAbs were used to analyze aspecific binding. Subsequently, the cells were washed with staining buffer and incubated (20 min) with the appropriate isotype-specific Alexa-647-, FITC- or PE- conjugated antibodies (Life Technologies). Cells were washed with staining buffer and with PBS and were resuspended in PBS. Data were acquired on a FACSCanto flow cytometer (Beckton Dickinson, Erembodegem, Belgium) with a minimum event count of 50 000 and analyzed with FACSDiva® software. Doublets were excluded based on FSC-H/FSC-A and SSC-H/SSC-A plots.

**Table 1:** **Monoclonal antibodies used in flow cytometry**

**Specificity**	**Clone**	**Isotype**	**Reference**
CD3	PTT3	IgG1	[27]
CD4	74-12-4	IgG2b	[28]
CD8α	11/295/33	IgG2a	[29]
CD8β	PG164A	IgG2a	[30]
CD21	BB6-11C9	IgG1	[30]
IgM	28.4.1	IgG1	[24]
MHCII	MSA3	IgG2a	[31]
SWC3a	74-22-15	IgG1	[28]
TCR1-N4 (δ chain)	PGBL22A	IgG1	[32]

### Cytokine secretion by PBMC and MC

PBMC and MC isolated from the spleen, pelvic lymph nodes and the *cervicales superficiales dorsales* lymph nodes were planted in 24-well microtiter plates at a concentration of 10^7^ cells/well. Subsequently, the cells were stimulated by adding 10^5^ viable purified S45 EBs to each well. Supernatant was collected 16, 24, 48 and 72 h later. The concentration of IL-1β, IL-4, IL-6, IL-8, IL-10, IL-12p40, IFN-γ and TNF-α in the supernatant was determined using commercially available ELISA kits (DuoSet® ELISA Development System, R&D Systems, Abingdon, UK). Assays were performed according to the manufacturer’s guidelines.

### Histopathology

Samples were fixed in 10% phosphate-buffered formalin, dehydrated and embedded in paraffin, sectioned at 5 μm and stained with haematoxylin and eosin. The samples were studied blindly. Slides were examined microscopically (Leitz). The microscopic findings were either graded (none (0), minimal (1), slight (2), moderate (3), marked (4) or severe (5) histological change) or indicated as present or absent without a grade.

### Statistical analysis

Statistical analyses were performed using SPSS 22 and results for all groups were compared by use of the non-parametric Mann–Whitney U test. Results were considered significantly different if *P* < 0.05.

## Results

### Macroscopic lesions

The mean scores for macroscopic lesions, determined at necropsy, are presented in Table 2. Macroscopic lesions were absent in all but one pig of the control group. One control animal had a severely inflamed vagina, cervix and uterus caused by an abnormal vaginal anatomy. Therefore, that animal was excluded from the experiment. All infected and re-infected animals showed gross pathology. The macroscopic lesions were generally more severe for the infection group than for the re-infection group. Four pigs (80%) of the infection group showed moderate to severe congestion of the genital tract (Additional file 1). The genital tract of the remaining pig (20%) of the infection group was slightly congested. In the re-infection group, all but one animal (80%) showed only slight congestion of the genital tract. In the other re-infected pig, a severely congested genital tract was noticed. In 60% of the pigs of the infection group, a large amount of clear watery exudate was present in almost the entire genital tract (Additional file 1), whereas only one animal (20%) of the re-infection group showed a moderate amount of watery exudate in the cervix. The pelvic lymph nodes were enlarged in 80% of the animals of both infected groups. Congestion of the pelvic lymph nodes could only be detected in four pigs (80%) of the infection group. Macroscopic lesions in the mesovarium and pelvic lymph nodes were significantly different between the groups.

**Table 2:** **Mean scores ± standard deviation (% of positive animals) for macroscopic lesions in the infection and the re-infection group at euthanasia** ^*^

**Tissue**	**Macroscopic lesion**	**Infection group**	**Re-infection group**
Vagina	Congestion	0.0 ± 0.00	0.2 ± 0.45 (20)
	Serous exudate	0.6 ± 1.34 (20)	0.0 ± 0.00
Cervix	Congestion	0.6 ± 1.34 (20)	0.2 ± 0.45 (20)
	Serous exudate	1.0 ± 1.41 (40)	0.4 ± 0.89 (20)
Uterus	Congestion	0.8 ± 1.10 (40)	1.0 ± 1.22 (60)
	Hypertrophy	0.6 ± 1.34 (20)	0.0 ± 0.00
	Serous exudate	1.6 ± 1.52 (60)	0.0 ± 0.00
Uterine tubes	Congestion	1.2 ± 1.10 (60)	0.4 ± 0.55 (40)
	Hypertrophy	0.6 ± 1.34 (20)	0.0 ± 0.00
	Serous exudate	1.2 ± 1.64 (40)	0.0 ± 0.00
Oviducts	Serous exudate	0.6 ± 1.34 (20)	0.0 ± 0.00
Ovaries	Congestion	0.4 ± 0.89 (20)	0.0 ± 0.00
*Lig. Latum uteri*	Congestion	0.8 ± 0.84 (60)	0.4 ± 0.55 (40)
Mesovarium	Congestion	1.6 ± 1.34 (80)^a^	0.2 ± 0.45 (20)
Mesosalpinx	Congestion	1.4 ± 1.52 (60)	0.2 ± 0.45 (20)
Spleen	Congestion	0.4 ± 0.89 (20)	0.0 ± 0.00
	Enlargement	0.0 ± 0.00	1.0 ± 1.41 (40)
Liver	Congestion	0.2 ± 0.45 (20)	0.0 ± 0.00
Pelvic lymph nodes	Congestion	1.6 ± 1.14 (80)^a,c^	0.0 ± 0.00^c^
	Enlargement	2.2 ± 1.3 (80)^a^	2.0 ± 1.22 (80)^b^
Cervical lymph node	Enlargement	1.8 ± 1.64 (60)	0.4 ± 0.89 (20)

^*^The mean score ± SD in the control group was 0.0 ± 0.00 for all tissues.

^a^ *P* < 0.05 for a comparison of the control group and the infection group.

^b^ *P* < 0.05 for a comparison of the control group and the re-infection group.

^c^ *P* < 0.05 for a comparison of the infection group and the re-infection group.

### *Chlamydia suis* vaginal excretion

**Figure 1:**
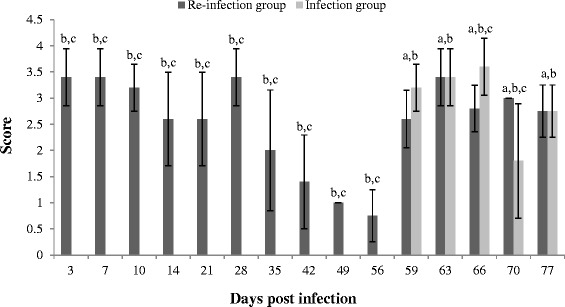
**Mean scores ± standard deviation for vaginal** ***C. suis*** **shedding from 3 to 77 dpi in the infection and the re-infection group.** The mean score ± SD in the control group was 0.0 ± 0.00 for all time points. ^a^ *P* < 0.05 for a comparison of the control group and the infection group. ^b^ *P* < 0.05 for a comparison of the control group and the re-infection group. ^c^ *P* < 0.05 for a comparison of the infection group and the re-infection group.

The presence of viable *C. suis* in vaginal swabs was examined using culture in McCoy cells. Figure 1 shows the culture scores for vaginal swabs collected at different time points post infection. All swabs taken at day 0 of the experiment were *Chlamydiaceae* negative. Vaginal swab samples of the control group remained negative throughout the experiment. Vaginal *C. suis* shedding was detected in the infection group from day 59, three days after infection of those animals, onwards. In the re-infection group, *C. suis* excretion was consistently high from day 3 to day 28 post primo-infection. After 28 dpi, mean culture scores decreased gradually until re-infection. Thereafter shedding was again consistently high. Before primo-infection of the infection group, the mean excretion scores of the re-infection group were statistically higher than those of the other groups. From day 59 onwards, vaginal shedding was significantly higher in the infection group and the re-infection group than in the control group. After primo-infection of the infection group, the mean culture scores of both infected groups did only differ statistically at day 66, when the infection group had the highest score, and at day 70, when the re-infection group had the highest score.

### Chlamydial antigen detection in the urogenital tract

Table 3 presents the mean scores for the presence of *Chlamydiaceae* in urogenital tissues collected at euthanasia, 21 days after infection or re-infection of the infection group and the re-infection group, respectively. All tissue samples from the non-infected pigs were negative, as were the spleen, the liver and the caecum of both infected groups. An ascending chlamydial infection occurred in all pigs of the infection group and in 4 of 5 pigs of the re-infection group, as chlamydial EBs and/or intracellular inclusions were detected in the upper genital tract of those animals. Intracellular chlamydial inclusions, indicating the presence of replicating chlamydiae, were detected in the urethra (20%), vagina (40%), cervix (60%), right uterine horn (20%), oviducts (20%) and ovaries (40%) of the infection group. In the re-infection group, chlamydial replication could only be observed in the urethra of one pig (20%), the vagina of two pigs (40%) and the right oviduct of one pig (20%). Overall, more chlamydial EBs and inclusions, demonstrated by higher mean scores, occurred in the urogenital tract of the infection group than of the re-infection group. Statistical analysis revealed significant differences between the mean scores of the right uterine horn and the left oviduct of both infected groups. Furthermore, mean scores for the presence of *Chlamydiaceae* in the vagina, right uterine horn, left oviduct and right ovary of the infection group were statistically higher than for the control group. In the re-infection group, the level of *Chlamydia* replication in the vagina and the urethra was significantly higher than in the non-infected pigs.

**Table 3:** **Mean scores ± standard deviation for the presence of** ***C. suis*** **in urogenital tissues of the infection and the re-infection group at euthanasia** *

**Tissue**	**Infection group**	**Re-infection group**
Vagina	1.80 ± 1.10^a^	2.00 ± 1.41^b^
Cervix	2.00 ± 1.87	0.40 ± 0.55
Corpus uteri	0.80 ± 0.45	0.20 ± 0.45
Uterine horn R	1.40 ± 0.89^a,c^	0.40 ± 0.55^c^
Uterine horn L	0.20 ± 0.45	0.40 ± 0.55
Oviduct R	0.80 ± 1.30	0.80 ± 1.30
Oviduct L	1.40 ± 0.89^a,c^	0.50 ± 0.58^c^
Ovary R	1.60 ± 1.34^a^	0.20 ± 0.45
Ovary L	0.80 ± 1.30	0.00 ± 0.00
Urethra	1.20 ± 1.30	1.20 ± 1.10^b^

*The mean score ± SD in the control group was 0.00 ± 0.00 for all tissues.

^a^ *P* < 0.05 for a comparison of the control group and the infection group.

^b^ *P* < 0.05 for a comparison of the control group and the re-infection group.

^c^ *P* < 0.05 for a comparison of the infection group and the re-infection group.

### Antibody responses

The *C. suis* S45-specific IgM, IgG and IgA antibody titers in serum (Table 4) and vaginal swab samples (Table 5) were determined following primo- and re-infection. The control pigs had no chlamydial antibodies in both serum and vaginal secretion throughout the experiment. Before infection, no serum or vaginal antibodies were detected in the animals of the infection and the re-infection group. Primo-infection induced IgM and IgG, but no IgA antibodies in serum and vaginal secretion. *C. suis* S45-specific serum IgM and IgG were observed from 7 days post primo-infection onwards and mean titers peaked at 14 or 21 dpi. Vaginal antichlamydial IgM and IgG antibodies appeared at 14 and at 14 and 21 days post primo-infection, respectively. Seven days after re-infection, mean *C. suis*-specific serum IgM and IgG titers had increased again and they kept on rising towards euthanasia. Antichlamydial mucosal IgM and IgG antibodies were detected from 7 to 14 and from 7 to 21 days post re-infection, respectively. Moreover, *C. suis*-specific serum and vaginal IgA antibodies appeared from 14 days following re-infection onwards. Compared to the antibody responses after primo-infection, re-infection with *C. suis* S45 induced lower IgM antibody titers and higher antichlamydial IgG and IgA titers, which is illustrative for a secondary antibody response.

**Table 4:** **Mean** ***C. suis*** **S45-specific IgM, IgG and IgA serum titers of the infection (I) and the re-infection (R) group ± standard deviation** ^*^

**Dpi**	**Procedure**	**Serum IgM**		**Serum IgG**		**Serum IgA**
		**Infection group**	**Re-infection group**	**Infection group**	**Re-infection group**	**Re-infection group**
0	Infection R	0 ± 0.00	0 ± 0.00	0 ± 0.00	0 ± 0.00	0 ± 0.00
7		0 ± 0.00^c^	30 ± 0.00^b,c^	0 ± 0.00^c^	48 ± 16.43^b,c^	0 ± 0.00
14		0 ± 0.00^c^	168 ± 98.59^b,c^	0 ± 0.00^c^	240 ± 0.00^b,c^	0 ± 0.00
21		0 ± 0.00^c^	60 ± 0.00^b,c^	0 ± 0.00^c^	144 ± 90.99^b,c^	0 ± 0.00
28		0 ± 0.00	0 ± 0.00	0 ± 0.00^c^	36 ± 13.42^b,c^	0 ± 0.00
35		0 ± 0.00	0 ± 0.00	0 ± 0.00^c^	48 ± 16.43^b,c^	0 ± 0.00
42		0 ± 0.00	0 ± 0.00	0 ± 0.00^c^	36 ± 13.42^b,c^	0 ± 0.00
49		0 ± 0.00	0 ± 0.00	0 ± 0.00^c^	54 ± 39.12^b,c^	0 ± 0.00
56	Infection I & R	0 ± 0.00	0 ± 0.00	0 ± 0.00^c^	48 ± 16.43^b,c^	0 ± 0.00
63		30 ± 0.00^a^	30 ± 0.00^b^	45 ± 21.21^a,c^	264 ± 131.45^b,c^	0 ± 0.00
70		216 ± 53.67^a,c^	42 ± 16.43^b,c^	120 ± 0.00^a,c^	336 ± 131.45^b,c^	30 ± 0.00^b,c^
77	Euthanasia	72 ± 26.83^a^	60 ± 0.00^b^	195 ± 178.12^a,c^	816 ± 321.99^b,c^	60 ± 0.00^b,c^

^*^The mean IgM, IgG and IgA serum antibody titers ± SD in the control group and the mean IgA serum titers ± SD in the infection group were 0 ± 0.00 for all time points. Antibody titers were defined as the reciprocal of the highest dilution that gave an absorbance reading at 405 nm above the cut-off value (mean absorbance of seronegative pig serum + twice the standard deviation).

^a^ *P* < 0.05 for a comparison of the control group and the infection group.

^b^ *P* < 0.05 for a comparison of the control group and the re-infection group.

^c^ *P* < 0.05 for a comparison of the infection group and the re-infection group.

**Table 5:** **Mean** ***C. suis*** **S45-specific IgM, IgG and IgA mucosal (vaginal) titers of the infection (I) and the re-infection (R) group ± standard deviation** ^*^

**Dpi**	**Procedure**	**Mucosal IgM**		**Mucosal IgG**		**Mucosal IgA**
		**Infection group**	**Re-infection group**	**Infection group**	**Re-infection group**	**Re-infection group**
0	Infection R	0 ± 0.00	0 ± 0.00	0 ± 0.00	0 ± 0.00	0 ± 0.00
7		0 ± 0.00	0 ± 0.00	0 ± 0.00	0 ± 0.00	0 ± 0.00
14		0 ± 0.00^c^	60 ± 0.00^b,c^	0 ± 0.00^c^	54 ± 13.42^b,c^	0 ± 0.00
21		0 ± 0.00	0 ± 0.00	0 ± 0.00^c^	30 ± 0.00^b,c^	0 ± 0.00
28-49		0 ± 0.00	0 ± 0.00	0 ± 0.00	0 ± 0.00	0 ± 0.00
56	Infection I & R	0 ± 0.00	0 ± 0.00	0 ± 0.00	0 ± 0.00	0 ± 0.00
63		0 ± 0.00^c^	30 ± 0.00^b,c^	0 ± 0.00^c^	48 ± 16.43^b,c^	0 ± 0.00
70		48 ± 16.43^a,c^	24 ± 13.42^b,c^	60 ± 0.00^a,c^	96 ± 32.86^b,c^	24 ± 13.42^b,c^
77	Euthanasia	0 ± 0.00	0 ± 0.00	96 ± 32.86^a,c^	48 ± 16.43^b,c^	30 ± 0.00^b,c^

^*^The mean IgM, IgG and IgA mucosal antibody titers ± SD in the control group and the mean IgA mucosal titers ± SD in the infection group were 0 ± 0.00 for all time points. Antibody titers were defined as the reciprocal of the highest dilution that gave an absorbance reading at 405 nm above the cut-off value (mean absorbance of seronegative pig serum + twice the standard deviation).

^a^ *P* < 0.05 for a comparison of the control group and the infection group.

^b^ *P* < 0.05 for a comparison of the control group and the re-infection group.

^c^ *P* < 0.05 for a comparison of the infection group and the re-infection group.

### Proliferative responses of PBMC and MC

Proliferative responses of PBMC to *C. suis* strain S45 were determined 7 and 10 days post primo-infection of the re-infection group, and again at day 63 and day 66, 7 and 10 days post infection or re-infection of the infection group and the re-infection group, respectively. At day 7 and 10, the proliferative activities of PBMC of all groups were statistically the same (data not shown). At day 63, the mean stimulation index (SI ± SD) of the PBMC for the control group, the infection group and the re-infection group were 2.03 ± 1.42, 2.15 ± 1.23 and 8.80 ± 5.46, respectively. The PBMC of the re-infection group showed significantly higher proliferative responses than the PBMC of the other groups. At day 66, the mean SI ± SD of the PBMC for the control, the infection and the re-infection group were 7.42 ± 2.87, 2.24 ± 1.35 and 7.29 ± 3.39. The proliferative activity of PBMC of the infection group was statistically lower than for the control group and the re-infection group. However, at day 66, the PBMC of the control group showed significantly higher proliferative responses to the positive control ConA, a T cell mitogen, than the PBMC of the other groups (data not shown). This indicates that the PBMC of the control group had a higher proliferative capacity than the other groups at that time and explains the high SI following stimulation with *C. suis* for the control group. At euthanasia, proliferative responses of MC from the spleen, cervical and pelvic lymph nodes were statistically the same for all groups (data not shown).

### Immune cell populations

Blood immune cell populations were analyzed by flow cytometry at 7 and 10 days post primo-infection of the re-infection group and again at day 63 and day 66, 7 and 10 days post infection or re-infection of the infection group and the re-infection group, respectively (Additional files 2 and 3). Seven days post primo-infection of the re-infection group, the infected animals had a significantly lower mean percentage of CD4^+^ T cells than the non-infected animals. At day 10, a significantly lower percentage of T cells was noticed in the re-infection group than in the infection group. At day 63, the re-infected animals had significantly higher mean percentages of T cells, B cells and monocytes, but a significantly lower mean percentage of pDC as compared to the control group or the infection group. Within total T cells, significantly less CD4^+^CD8^−^ T cells were present in the re-infection group than in the infection group. At day 66, the re-infection group had significantly higher percentages of B cells and CD4^−^CD8^+^ T cells. At that time, a significantly lower percentage of monocytes was detected in the infection group than in the other groups. Furthermore, immune cell populations in the spleen, cervical and pelvic lymph nodes were analyzed at euthanasia (Additional files 2 and 3). For the spleen, the mean percentage of T cells was significantly lower in the re-infection group than in the other groups and within total T cells significantly less CD4^+^CD8^+^ and CD4^−^CD8^+^ T cells and significantly more CD4^+^CD8^−^ T cells were present in the re-infection group. Moreover, the re-infection group had significantly lower mean percentages of B cells and monocytes in the spleen as compared to the control group, but a significantly higher percentage of CD3^−^CD4^+^CD8^−^ spleen MC, which contain pDC, than the other groups. The mean percentage of NK cells in the spleen was significantly higher for the infection group. For the cervical lymph nodes, significantly higher mean percentages of CD4^+^CD8^−^ T cells, B cells and monocytes, besides a significantly lower mean percentage of CD3^−^CD4^+^CD8^−^ MC, containing pDC, were noticed in the non-infected animals. For the pelvic lymph nodes, the control pigs had significantly higher mean percentages of γδ T cells, IgM^+^ B cells and monocytes. The mean percentage of NK cells was significantly higher for the infection group than for the re-infected animals. Within total T cells, significantly more CD4^−^CD8^+^ T cells were detected in the re-infection group than in the other groups.

### Cytokine secretion by PBMC and MC

Results for cytokine detection at 16 h and 72 h post stimulation are presented in Additional files 4 and 5. Data of cytokine production at 24 h and 48 h post stimulation are not shown, since the results were either without statistical differences between the groups or with statistical differences similar to these observed at 72 h post stimulation. Compared to the amount of cytokines produced at 16 h following stimulation, PBMC and MC had produced generally more IFN-γ, TNF-α, IL-6 and IL-10 and less IL-1β at 72 h after stimulation. Only the data of cytokine secretion at 72 h post stimulation are discussed in detail.

At 7 dpi, the PBMC of the re-infection group produced significantly more TNF-α, IL-1β, IL-6 and IL-12p40 than the PBMC of the other groups. At day 10, cytokine production by PBMC was statistically the same for all groups. At day 63, seven days post infection or re-infection of the infection group and the re-infection group, respectively, the mean concentrations of IFN-γ, TNF-α, IL-1β, IL-6, IL-10 and IL-12p40 for the re-infection group were significantly higher than the concentrations produced by the PBMC of the other groups. At day 66, the PBMC of the re-infected animals had produced significantly more TNF-α, IL-1β, IL-6 and IL-10 than the PBMC of the other animals. Furthermore, significantly more IL-8 and IL-12p40 was produced by the blood mononuclear cells from the re-infection group when compared to the infection group. For the infection group, mean IL-1β and IL-10 concentrations in PBMC culture media were significantly lower than for the control group.

At euthanasia, spleen MC of the infection group had produced significantly less IFN-γ, TNF-α, IL-1β, IL-6, IL-8 and IL-12p40 than MC of the other groups. The production of TNF-α, IL-6 and IL-8 by cervical lymph node MC was significantly higher for the control animals than for the infection or the re-infection group. Mean IL-6 amounts produced by MC isolated from the pelvic lymph nodes were significantly higher for the control group than for both infected groups. The MC from pelvic lymph nodes of the infection group produced significantly less IL-4 than the MC of the control group.

### Histopathology

An overview of the mean scores of the histopathological lesions found in the urogenital tract of the pigs at euthanasia is presented in Table 6. Histopathological findings consisted of: (a) intraluminal proteinaceous fluid; (b) the presence of a superficial layer of exfoliated (epithelial) cells and/or inflammatory cells: cells and debris laying on the surface of the epithelium; (c) interepithelial inflammatory cells, thus indicating the presence of migrating lymphocytes or polymorphonuclear cells in the epithelium; (d) degeneration (vacuolation) and/or apoptosis (cell death) of epithelial cells; (e) infiltration of inflammatory cells in the lamina propria with infiltration of the connective tissue layer beneath the epithelium with polymorphonuclear cells (pmc), a feature of more acute inflammation, or with mononuclear cells (mnc) (lymphocytes and/or plasma cells), a feature of more subacute to chronic inflammation; (f) oedema in the lamina propria: presence of loosely arranged fibers in the connective tissue and/or eosinophilic fluid between the fibers; (g) infiltration of inflammatory cells (pmc or mnc) in the muscular layer, (h) or in the serosa. In the corpus uteri, both uterine horns and the right oviduct mononuclear inflammatory cells increased slightly in the mucosa with infection with *C. suis* S45 (infection group) and even more when animals were re-infected (re-infection group). The inflammatory cells in the infection group were more periglandularly clustered in the deep mucosa, whereas in the re-infection group the inflammation was more diffusely present throughout the mucosa. No lesions were noted in both ovaries and the lesions observed in the left oviduct, the cervix, the vagina and the urethra did not differ statistically between the different groups. In the liver of two pigs of the re-infection group slight to moderate, focal to multifocal granulomatous inflammations within the portal areas were noticed. The same animals presented also minimal to slight diffuse follicular hyperplasia in the spleen, a feature of immunological stimulation. The spleen and liver samples from the remaining pigs and all caecum samples showed no histopathological changes.

**Table 6:** **Mean scores ± standard deviations for histopathological findings** ^**#**^

**Tissue**	**Histopathological findings**	**Control group**	**Infection group**	**Re-infection group**
Vagina	Exfoliation*	0.5 ± 1.00	0.2 ± 0.45	0.0 ± 0.00
	Interepithelial inflammatory cells	0.0 ± 0.00	0.4 ± 0.55	0.2 ± 0.45
	Degeneration and/or apoptosis of epithelial cells	0.0 ± 0.00	0.4 ± 0.55	0.4 ± 0.55
	Infiltration of mnc in the lamina propria	1.5 ± 0.58	1.4 ± 0.55	1.2 ± 0.45
Cervix	Interepithelial inflammatory cells	1.0 ± 0.82	0.8 ± 0.84	1.2 ± 0.84
	Degeneration and/or apoptosis of epithelial cells	0.0 ± 0.00	0.2 ± 0.45	0.0 ± 0.00
	Infiltration of mnc in the lamina propria	2.0 ± 0.82	1.4 ± 0.55	1.8 ± 0.84
Corpus	Interepithelial inflammatory cells	1.0 ± 0.00	1.2 ± 0.45	1.2 ± 0.45
uteri	Degeneration and/or apoptosis of epithelial cells	0.3 ± 0.50	0.2 ± 0.45	0.0 ± 0.00
	Infiltration of mnc in the lamina propria	1.0 ± 0.00^b^	1.2 ± 1.30	2.0 ± 0.71^b^
	Oedema lamina propria	2.3 ± 0.96	1.8 ± 1.30	1.4 ± 1.34
Uterine	Exfoliation*	0.0 ± 0.00	0.0 ± 0.00	0.4 ± 0.89
Horn R	Interepithelial inflammatory cells	1.3 ± 0.50	1.2 ± 0.45	1.2 ± 0.45
	Degeneration and/or apoptosis of epithelial cells	0.3 ± 0.50	0.4 ± 0.55	0.0 ± 0.00
	Infiltration of mnc in the lamina propria	1.3 ± 0.50^b^	2.2 ± 1.10	2.6 ± 0.55^b^
	Oedema lamina propria	1.5 ± 0.58	1.6 ± 1.34	1.2 ± 1.30
Uterine	Exfoliation*	0.0 ± 0.00	0.4 ± 0.89	0.0 ± 0.00
Horn L	Interepithelial inflammatory cells	1.8 ± 0.50	1.8 ± 0.45	1.2 ± 0.45
	Degeneration and/or apoptosis of epithelial cells	0.3 ± 0.50	0.8 ± 0.45	0.8 ± 0.45
	Infiltration of mnc in the lamina propria	1.3 ± 0.50^b^	2.2 ± 1.10	2.4 ± 0.55^b^
	Oedema lamina propria	1.0 ± 0.82	1.4 ± 0.55	1.0 ± 0.00
Oviduct R	Intraluminal proteinaceous fluid	4 of 4	4 of 5	4 of 5
	Exfoliation*	0.5 ± 0.58	0.0 ± 0.00	0.0 ± 0.00
	Infiltration of mnc in the lamina propria	1.0 ± 0.00^b^	1.4 ± 0.55^c^	2.2 ± 0.45^b,c^
Oviduct L	Intraluminal proteinaceous fluid	1 of 4	1 of 5	2 of 5
	Exfoliation*	0.3 ± 0.50	0.2 ± 0.45	0.4 ± 0.55
	Interepithelial inflammatory cells	0.0 ± 0.00	0.4 ± 0.55	0.6 ± 0.55
	Infiltration of pmc in the lamina propria	0.3 ± 0.50	0.0 ± 0.00	0.0 ± 0.00
	Infiltration of mnc in the lamina propria	1.3 ± 0.50	1.4 ± 0.89	1.4 ± 0.55
Urethra	Interepithelial inflammatory cells	0.3 ± 0.50	0.2 ± 0.45	0.0 ± 0.00
	Infiltration of mnc in the lamina propria	1.0 ± 0.00	0.8 ± 1.30	1.4 ± 1.52

^#^The mean scores ± SD for the histopathological parameters which are not shown in the table, were 0.0 ± 0.00 for the three groups.

*Superficial layer of exfoliated (epithelial) cells and/or inflammatory cells.

pmc: polymorphonuclear inflammatory cells; mnc: mononuclear inflammatory cells.

^b^P < 0.05 for a comparison of the control group and the re-infection group.

^c^P < 0.05 for a comparison of the infection group and the re-infection group.

## Discussion

Genital *C. suis* infections are likely to be transmitted venereally, since these bacteria have been detected in the semen of boars [1,12,13]. However, non-sexual transmission cannot be ruled out, for instance by fecal contamination of the vagina. In fact, *C. suis* has been commonly detected in the gastrointestinal tract of pigs [33], and in most of the cases it is associated with subclinical infections. Therefore, the gastrointestinal tract could serve as a reservoir for chlamydial genital tract infection [34]. In this study, female pigs were successfully infected in the genital tract with *C. suis* strain S45, which was isolated from feces of an asymptomatic pig. This indeed suggests that persistent intestinal *C. suis* infections are able to cause pathology in the genital tract of pigs.

In the present study, all infected animals showed gross pathology at necropsy. However, the pigs that were infected once had significantly more severe lesions than the re-infected animals. Moreover, more chlamydial EBs and inclusions were found in the urogenital tract of the infection group than in the re-infected pigs. For the infection group, chlamydial replication occurred throughout the urogenital tract and even in the ovaries, whereas for the re-infection group, inclusions were scarce. This indicates that a certain level of protection against re-infection was developed following primo-infection. This presumption is in accordance with human epidemiologic studies suggesting that prior genital infection with *Chlamydia trachomatis*, which is phylogenetically highly related to *C. suis*, confers some short term protection against re-infection [35,36]. Moreover, in all evaluated animal models of genital chlamydial infection, including the mouse [37], guinea pig [38] and macaque [39], it has been demonstrated that at least partial immunity to re-infection develops. Nevertheless, vaginal inoculation with *C. suis* resulted in an ascending infection in both groups. Likewise, *C. trachomatis* was able to ascend in the genital tract of pigs after intravaginal inoculation [40]. No chlamydial bacteria were detected outside the urogenital tract. With the exception of the lymphogranuloma venereum strains, *C. trachomatis* also does not disseminate beyond the urogenital tract [41].

Infection of the female porcine genital tract with *C. suis* resulted microscopically in a slight increase of mononuclear inflammatory cells in the mucosa of the corpus uteri, both uterine horns and the right oviduct. Surprisingly, this histopathological finding was even more prominent when animals were re-infected, which is not in accordance with the less severe macroscopic lesions and the lower mean scores for the presence of chlamydial bacteria observed in this group. However, these mononuclear cells, including lymphocytes and plasma cells, may indicate the mobilization of memory cells in response to re-infection and can contribute to protective immunity against infection. In the future, we will include immunohistochemistry to obtain in situ information on the cells contributing to this partial protection. Schautteet et al. [42] also observed that protection against gross lesions was correlated with a higher infiltration of mononuclear inflammatory cells in the genital tract mucosa of pigs intravaginally infected with *C. trachomatis*.

Vaginal chlamydial excretion was observed in all infected animals from three dpi onwards. There were no differences noticed between shedding after primo-infection and re-infection. Interestingly, vaginal excretion started to decrease gradually at four weeks post primo-infection and one out of five animals stopped shedding chlamydial bacteria at 56 dpi. Since the animals were infected again at that moment, we could not determine whether the pigs were able to resolve a genital *C. suis* infection spontaneously after a certain period of time. Also, it would have been interesting to follow up the animals for a longer period than three weeks post re-infection in order to assess whether chlamydial excretion stops earlier post re-infection than post primo-infection.

Primo-infection with *C. suis* induced antichlamydial IgM and IgG, but no IgA antibodies in sera and vaginal secretions. Compared to the antibody responses after primo-infection, re-infection with *C. suis* resulted in lower IgM antibody titers and higher IgG and IgA titers in sera and genital secretions, which is illustrative for a secondary antibody response. Furthermore, re-infection was, in contrast to primo-infection, able to induce *C. suis*-specific proliferation of PBMC after 7 and 10 days, which indicates the mobilization of memory cells in response to re-infection. Re-infection was associated with a shift to more B cells, monocytes and CD4^−^CD8^+^ T cells, but less pDC and CD4^+^CD8^−^ T cells within PBMC. However, the immune responses detected in the blood do not necessarily reflect the immune responses induced at the local site of infection. Again, immunohistochemistry might assist in determining the local responses. The shifts in immune cell populations from the spleen, cervical and pelvic lymph nodes are presumably not resulting from *C. suis*-specific MC since their proliferative responses were not statistically different between groups.

Primary genital *C. suis* infection of the re-infection group (nine-week-old pigs) resulted in increased secretion of TNF-α, IL-1β, IL-6 and IL-12p40 by PBMC, whereas primo-infection of the infection group (seventeen-week-old animals) decreased the IL-1β, IL-8 and IL-10 secretion by PBMC. This discrepancy could be due to age differences between both groups at the moment of the primo-infection. In pigs, various aspects of the immune system change with age such as the level of NK cells, which was shown to be higher in younger pigs [43,44]. Since NK cells produce cytokines like TNF-α, this can explain the higher cytokine production following primo-infection at nine weeks of age. Furthermore, it was demonstrated that neonatal porcine blood DCs were more responsive to stimulation with toll-like receptor ligands than adult porcine blood DCs, since they showed higher expression levels of cytokines and chemokines following stimulation [45]. After re-infection, higher concentrations of IFN-γ and IL-10 were produced by PBMC, next to higher amounts of the cytokines upregulated after primo-infection.

Our data suggest that antibody producing B cells, mononuclear phagocytes, CD8^+^ T cells, and IFN-γ may contribute to protection against a genital *C. suis* infection. The role of IL-10 is less clear. Antibodies, and especially mucosal antibodies, albeit not considered as crucial, may contribute to protection by neutralization and opsonization of extracellular *Chlamydia*. Indeed, antibodies clearly play an important role in resistance to genital *Chlamydia muridarum* re-infection in mice [46]. Moreover, mucosal antichlamydial IgA antibodies have been associated with the resolution of *C. muridarum* and *C. trachomatis* infection in mice and women, respectively [47,48]. The Th1-type cytokine IFN-γ was also shown to be essential for the resolution of *C. muridarum* infection [49,50]. IFN-γ produced by NK cells, CD4^+^ Th1 cells and CD8^+^ cytotoxic T cells is known to up-regulate and/or induce expression of major histocompatibility complex (MHC) class I and II molecules on a number of cells, including antigen presenting cells. In addition, IFN-γ favours immunoglobulin class switching to the IgG2a isotype and activation of cytotoxic T lymphocytes, which induce apoptosis in infected cells. IL-10, as the main anti-inflammatory cytokine, plays important roles in immune-homeostasis after microbe elimination [51]. IL-10 expression results in an inhibition of antigen presentation and MHCII expression on the surface of infected cells. However, as an anti-inflammatory cytokine, it also deactivates macrophages and interferes with the Th1 response through inhibition of the NF-κB pathway [52]. Interestingly, Moore-Connors et al. [53] showed that *C. muridarum* induces a novel CD43^−^CD1d^hi^CD5^+^ IL-10-producing regulatory B cell population (Bregs) during a genital infection in mice. These IL-10 producing B cells displayed *bona fide* regulatory Breg activity by potently suppressing IFN-γ production in vitro in an IL-10-dependent manner. We do not know if these IL-10 producing CD43^−^CD1d^hi^CD5^+^ Bregs do exist in the pig and if they can be induced by *C. suis*, but it could be a possible explanation for our observation that CD4^+^ Th1 cells, which are generally considered as crucial immune effectors in antichlamydial immunity, were less dominantly present.

In conclusion, we demonstrated that *C. suis* strain S45 is pathogenic for the female porcine urogenital tract. Chlamydial replication occurred throughout the urogenital tract, causing inflammation and pathology. Furthermore, genital infection elicited both cellular and humoral immune responses. Compared to the primo-infection of pigs with *C. suis*, re-infection was characterized by less severe macroscopic lesions and significantly less chlamydial EBs and inclusions in the urogenital tract. This indicates the development of a certain degree of protection following the initial infection. Protective immunity against re-infection coincided with higher antichlamydial IgG and IgA antibody titers in sera and vaginal secretions, higher proliferative responses of peripheral blood mononuclear cells (PBMC), higher percentages of blood B lymphocytes, monocytes and CD8^+^ T cells and upregulated production of IFN-γ and IL-10 by PBMC.

## Additional files

Additional file 1:
**Macroscopic lesions in an animal from the infection group.** Additional file 1 shows the most prominent gross pathology in the infection group at euthanasia. (A) Congestion of the *lig. latum uteri* (arrow a) and the mesovarium and mesosalpinx (arrow b). A large amount of clear watery exudate was present in the lumen of the vagina, cervix, corpus uteri (arrow c) and uterine tubes. The uterine tubes were severely dilated by the presence of the exudate in their lumen (arrow d). (B) Congestion of the mesovarium and mesosalpinx (arrow e). Hyperemia of the uterine tube (arrow f). The mucosa of the uterine tube is congested and oedematous and serous exudate was present in the lumen (arrow g). Additional file 2:
**Mean percentage ± standard deviation of different immune cell populations within PBMC, isolated at 7 and 10 days post infection or re-infection, and in spleen, cervical and pelvic lymph nodes at euthanasia.** Additional file 2 presents the mean percentages of total T cells, γδ T cells, mature and IgM^+^ B cells, monocytes, NK cells and plasmacytoid dendritic cells in the blood at different time points post infection and in the spleen, cervical and pelvic lymph nodes at euthanasia. Additional file 3:
**Mean percentage of different subpopulations within total T cells (CD3** ^**+**^ **) in the blood, isolated at 7 and 10 days post infection or re-infection, and in spleen, cervical and pelvic lymph nodes at euthanasia.** Additional file 3 shows the mean percentages of CD4^+^CD8^−^, CD4^−^CD8^+^, CD4^+^CD8^+^ and CD4^−^CD8^−^ subpopulations within total T cells in the blood at different time points post infection and in the spleen, cervical and pelvic lymph nodes at euthanasia. Additional file 4:
**Mean cytokine concentration (pg/mL) ± standard deviation in culture medium of PBMC, isolated at 7 and 10 days post infection or re-infection, and of spleen, pelvic and cervical lymph node MC at euthanasia, measured at 16 h post stimulation with** ***C. suis*** **S45.** Additional file 4 presents the results for cytokine detection at 16 h post stimulation. Additional file 5:
**Mean cytokine concentration (pg/mL) ± standard deviation in culture medium of PBMC, isolated at 7 and 10 days post infection or re-infection, and of spleen, pelvic and cervical lymph node MC at euthanasia, measured at 72 h post stimulation with** ***C. suis*** **S45.** Additional file 5 presents the results for cytokine detection at 72 h post stimulation.
