# Supplementary figures and images for: The immune response against Chlamydia suis genital tract infection partially protects against re-infection
Source: Vet Res. 2014 Sep 25;45(1):95. doi: 10.1186/s13567-014-0095-6 (PMC4181727; doi:10.1186/s13567-014-0095-6)

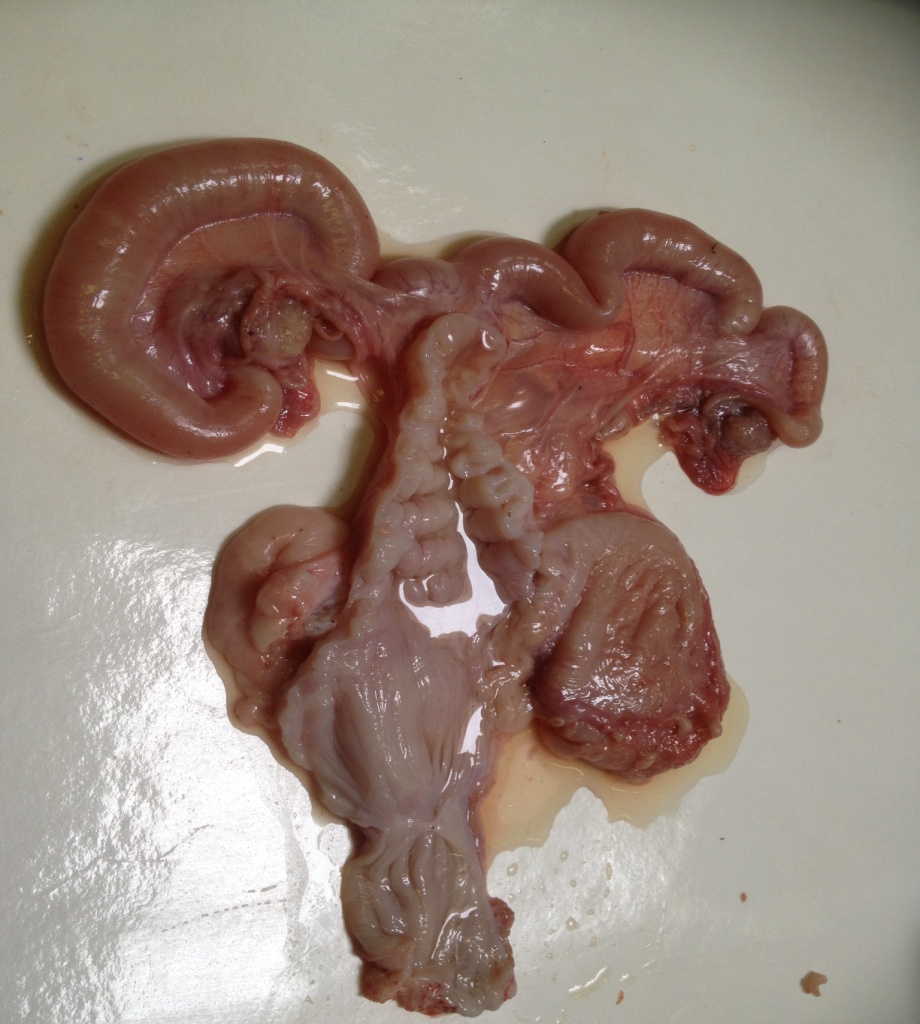

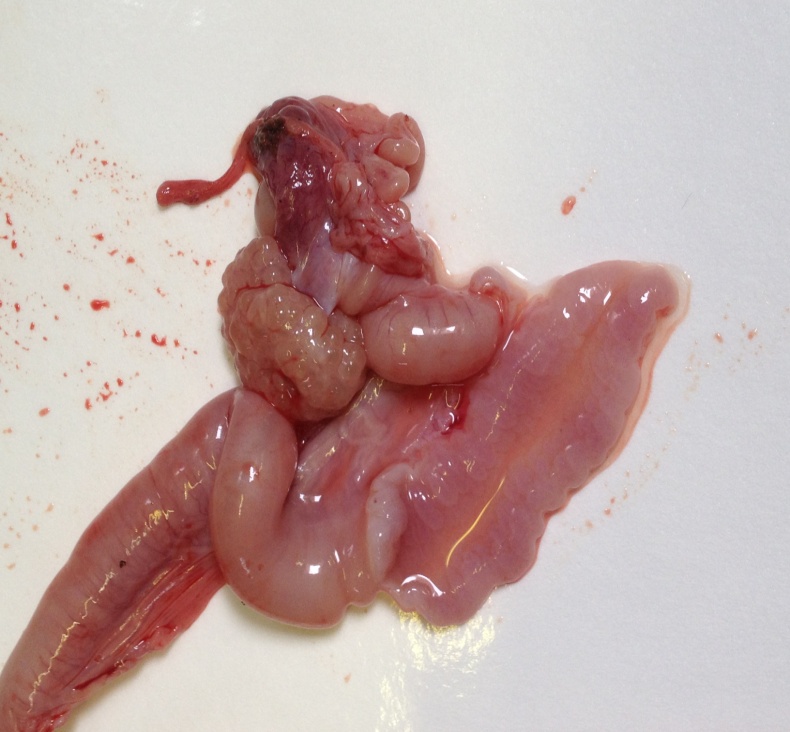


g

e

f

**B**

b

a

d

c

**A**

Supplement: Additional file 1: — Macroscopic lesions in an animal from the infection group. Additional file 1 shows the most prominent gross pathology in the infection group at euthanasia. (A) Congestion of the lig. latum uteri (arrow a) and the mesovarium and mesosalpinx (arrow b). A large amount of clear watery exudate was present in the lumen of the vagina, cervix, corpus uteri (arrow c) and uterine tubes. The uterine tubes were severely dilated by the presence of the exudate in their lumen (arrow d). (B) Congestion of the mesovarium and mesosalpinx (arrow e). Hyperemia of the uterine tube (arrow f). The mucosa of the uterine tube is congested and oedematous and serous exudate was present in the lumen (arrow g). [file 13567_2014_95_MOESM1_ESM.docx]
