# Supplementary material for: The immune response against Chlamydia suis genital tract infection partially protects against re-infection
Source: Vet Res. 2014 Sep 25;45(1):95. doi: 10.1186/s13567-014-0095-6 (PMC4181727; doi:10.1186/s13567-014-0095-6)
Supplement: Additional file 2: — Mean percentage ± standard deviation of different immune cell populations within PBMC, isolated at 7 and 10 days post infection or re-infection, and in spleen, cervical and pelvic lymph nodes at euthanasia. Additional file 2 presents the mean percentages of total T cells, γδ T cells, mature and IgM+ B cells, monocytes, NK cells and plasmacytoid dendritic cells in the blood at different time points post infection and in the spleen, cervical and pelvic lymph nodes at euthanasia. [file 13567_2014_95_MOESM2_ESM.docx]

|  | Group ♯ | Total  T cells * | γδ T cells * | Mature  B cells * | IgM^+^  B cells * | Monocytes* | NK cells * | pDC ** |
| --- | --- | --- | --- | --- | --- | --- | --- | --- |
| PBMC  Day 7  (7 dpi 1 R) | C | 46.40 ± 5.23 | 17.60 ± 3.70 | 11.40 ± 6.92 | 12.10 ± 7.24 | 1.63 ± 0.38 | 10.5 ± 4.59 | 0.43 ± 0.21 |
|  | I | 47.38 ± 5.87 | 17.50 ± 4.23 | 11.40 ± 4.60 | 10.96 ± 2.95 | 1.72 ± 1.45 | 11.16 ± 3.49 | 0.38 ± 0.16 |
|  | R | 46.46 ± 10.72 | 18.56 ± 5.10 | 10.10 ± 2.84 | 11.20 ± 2.30 | 1.38 ± 0.19 | 11.64 ± 4.72 | 0.26 ± 0.13 |
|  |  |  |  |  |  |  |  |  |
| PBMC  Day 10  (10 dpi 1 R) | C | 47.25 ± 5.79 | 19.70 ± 3.93 | 12.03 ± 5.71 | 12.73 ± 6.18 | 2.20 ± 0.76 | 6.63 ± 2.93 | 0.58 ± 0.17 |
|  | I | 52.02 ± 6.43^a^ | 17.54 ± 6.36 | 10.70 ± 4.77 | 10.54 ± 5.98 | 2.06 ± 0.80 | 11.66 ± 6.00 | 0.46 ± 0.28 |
|  | R | 42.53 ± 4.86^b^ | 21.75 ± 7.07 | 15.53 ± 2.76 | 16.53 ± 2.64 | 1.93 ± 0.56 | 6.78 ± 3.04 | 0.40 ± 0.14 |
|  |  |  |  |  |  |  |  |  |
| PBMC  Day 63  (7 dpi 1 I;  7 dpi 2 R) | C | 45.23 ± 11.23 | 25.50 ± 5.20 | 4.35 ± 2.41^a^ | 4.05 ± 2.20^a^ | 1.08 ± 0.60^a^ | 4.35 ± 2.37 | 1.28 ± 0.75^a^ |
|  | I | 32.48 ± 19.11^a^ | 25.00 ± 9.40 | 4.56 ± 4.96 | 4.00 ± 4.62 | 1.14 ± 1.29 | 4.32 ± 0.98 | 1.64 ± 0.87 |
|  | R | 59.98 ± 9.02^b^ | 30.83 ± 7.65 | 7.86 ± 2.42^b^ | 9.10 ± 2.30^b^ | 2.28 ± 0.38^b^ | 5.44 ± 3.40 | 0.46 ± 0.19^b^ |
|  |  |  |  |  |  |  |  |  |
| PBMC  Day 66  (10 dpi 1 I;  10 dpi 2 R) | C | 62.50 ± 5.13 | 31.83 ± 5.99 | 5.68 ± 1.79^a^ | 6.30 ± 1.99^a^ | 2.10 ± 0.48^a^ | 2.75 ± 1.34 | 0.88 ± 0.56 |
|  | I | 55.92 ± 11.01 | 24.90 ± 3.43 | 4.80 ± 3.14^a^ | 4.54 ± 2.81^a^ | 0.68 ± 0.81^b^ | 5.52 ± 2.38 | 0.78 ± 0.43 |
|  | R | 55.50 ± 4.63 | 29.58 ± 7.03 | 13.66 ± 2.01^b^ | 13.38 ± 2.28^b^ | 1.94 ± 0.40^a^ | 4.03 ± 2.48 | 0.50 ± 0.08 |
|  |  |  |  |  |  |  |  |  |
| Spleen  Day 77  (21 dpi 1 I;  21 dpi 2 R) | C | 55.75 ± 4.40^a^ | 29.33 ± 9.67 | 10.90 ± 3.10^a^ | 9.38 ± 2.71^a^ | 8.48 ± 1.54^a^ | 4.78 ± 2.59 | 4.25 ± 1.22^a^ |
|  | I | 59.65 ± 4.78^a^ | 26.45 ± 3.00 | 7.88 ± 4.27 | 6.72 ± 3.82 | 5.30 ± 3.57 | 7.93 ± 2.93^a^ | 4.35 ± 1.11^a^ |
|  | R | 25.66 ± 12.26^b^ | 24.46 ± 6.47 | 4.54 ± 1.96^b^ | 4.56 ± 2.25^b^ | 3.36 ± 1.82^b^ | 3.28 ± 0.73^b^ | 6.66 ± 1.29^b^ |
|  |  |  |  |  |  |  |  |  |
| Cervical LN Day 77  (21 dpi 1 I;  21 dpi 2 R) | C | 48.18 ± 9.81 | 17.78 ± 9.58 | 20.43 ± 9.53^a^ | 17.55 ± 8.27^a^ | 2.13 ± 1.05^a^ | 1.20 ± 0.94 | 0.20 ± 0.08^a^ |
|  | I | 26.47 ± 19.44 | 11.30 ± 6.35 | 0.40 ± 0.36^b^ | 0.17 ± 0.15^b^ | 0.53 ± 0.42^b^ | 6.83 ± 5.24 | 2.00 ± 2.08^b^ |
|  | R | 21.73 ± 25.17 | 9.27 ± 7.42 | 0.33 ± 0.32^b^ | 0.07 ± 0.12^b^ | 0.60 ± 0.53 | 3.70 ± 3.32 | 1.23 ± 0.35^b^ |
|  |  |  |  |  |  |  |  |  |
| Pelvic LN  Day 77  (21 dpi 1 I;  21 dpi 2 R) | C | 35.58 ± 13.77 | 18.98 ± 3.81^a^ | 15.85 ± 7.10 | 18.1 ± 5.99^a^ | 4.10 ± 1.68^a^ | 2.83 ± 1.23 | 1.35 ± 0.81 |
|  | I | 39.36 ± 15.28 | 14.92 ± 2.90^b^ | 14.66 ± 6.33 | 8.00 ± 1.29^b^ | 2.20 ± 0.60^b^ | 3.96 ± 1.53^a^ | 1.14 ± 0.51 |
|  | R | 33.86 ± 18.49 | 11.24 ± 4.58^b^ | 7.88 ± 4.38 | 6.08 ± 2.60^b^ | 2.16 ± 1.57 | 1.56 ± 0.46^b^ | 1.02 ± 0.36 |

^a,b,c^ For each time point or tissue, means with different superscripts within a column are significantly different (*P* < 0.05).

♯ C: control group; I: infection group; R: re-infection group

* Total T cells: CD3^+^; γδ T cells: CD8β^-^ TCR1-N4^+^; Mature B cells: MHCII^+^ CD21^+^; IgM^+^ B cells (immature and mature naive B cells): MHCII^+^ IgM^+^; Monocytes: MHCII^+^ SWC3^+^; NK cells: CD3^-^ CD4^-^ CD8^+^; pDC (plasmacytoid dendritic cells): CD3^-^ CD4^+^ CD8^-^

** For the lymphoid tissues the CD3^-^CD4^+^CD8^-^ population does not exist entirely of pDC, but also contains an unknown lineage, presumably of myeloid origin.
