# Supplementary material for: The immune response against Chlamydia suis genital tract infection partially protects against re-infection
Source: Vet Res. 2014 Sep 25;45(1):95. doi: 10.1186/s13567-014-0095-6 (PMC4181727; doi:10.1186/s13567-014-0095-6)
Supplement: Additional file 3: — Mean percentage of different subpopulations within total T cells (CD3 + ) in the blood, isolated at 7 and 10 days post infection or re-infection, and in spleen, cervical and pelvic lymph nodes at euthanasia. Additional file 3 shows the mean percentages of CD4+CD8−, CD4−CD8+, CD4+CD8+ and CD4−CD8− subpopulations within total T cells in the blood at different time points post infection and in the spleen, cervical and pelvic lymph nodes at euthanasia. [file 13567_2014_95_MOESM3_ESM.docx]

T cells were divided into four subpopulations based on the expression of CD4 and CD8.

^a,b,c^ For each time point or tissue, different letters within a data series are significantly different (*P* < 0.05). C: control group; I: infection group; R: re-infection group.
