# Supplementary material for: The immune response against Chlamydia suis genital tract infection partially protects against re-infection
Source: Vet Res. 2014 Sep 25;45(1):95. doi: 10.1186/s13567-014-0095-6 (PMC4181727; doi:10.1186/s13567-014-0095-6)
Supplement: Additional file 4: — Mean cytokine concentration (pg/mL) ± standard deviation in culture medium of PBMC, isolated at 7 and 10 days post infection or re-infection, and of spleen, pelvic and cervical lymph node MC at euthanasia, measured at 16 h post stimulation with C. suis S45. Additional file 4 presents the results for cytokine detection at 16 h post stimulation. [file 13567_2014_95_MOESM4_ESM.docx]

|  | Group * | IFN-γ | TNF-α | IL-1β | IL-4 | IL-6 | IL-8 | IL-10 | IL-12p40 |
| --- | --- | --- | --- | --- | --- | --- | --- | --- | --- |
| PBMC  Day 7  (7 dpi 1 R) | C | 14.9 ± 18.6 | 209.2 ± 241.5^a^ | 6775.7 ± 3002.1^a^ | 134.7 ± 72.8 | 351.6 ± 333.8^a^ | 23508.0 ± 12568.8 | 606.7 ± 142.9 | 387.5 ± 90.5^a^ |
|  | I | 61.3 ± 60.0 | 242.0 ± 105.0^a^ | 8035.1 ± 2768.7 | 107.9 ± 68.3 | 408.3 ± 211.9^a^ | 32039.6 ± 10677.3 | 554.3 ± 83.2 | 383.4 ± 94.7^a^ |
|  | R | 1916.1 ± 1326.0 | 781.8 ± 458.0^b^ | 11309.1 ± 2917.9^b^ | 120.1 ± 125.2 | 1241.4 ± 728.5^b^ | 30800.6 ± 11017.7 | 994.7 ± 476.8 | 719.8 ± 157.8^b^ |
|  |  |  |  |  |  |  |  |  |  |
| PBMC  Day 10  (10 dpi 1 R) | C | 100.3 ± 193.0 | 668.0 ± 1132.5 | 5532.0 ± 2827.4 | 107.7 ± 41.3 | 377.4 ± 648.7 | 19573.9 ± 15280.0 | 642.7 ± 392.8 | 424.4 ± 96.2 |
|  | I | 57.5 ± 70.2 | 419.1 ± 458.1 | 4731.6 ± 2143.3 | 103.1 ± 17.9 | 415.1 ± 296.9 | 16866.9 ± 9817.5 | 621.8 ± 171.5 | 458.5 ± 238.3 |
|  | R | 272.4 ± 263.2 | 668.7 ± 411.1 | 6940.0 ± 3052.6 | 120.6 ± 32.5 | 642.5 ± 479.6 | 25847.5 ± 10291.6 | 742.7 ± 160.2 | 617.6 ± 172.8 |
|  |  |  |  |  |  |  |  |  |  |
| PBMC  Day 63  (7 dpi 1 I;  7 dpi 2 R) | C | 0.0 ± 0.0 | 103.9 ± 89.4^a^ | 4231.3 ± 4012.4 | 103.7 ± 14.7 | 2.2 ± 4.4 | 4916.9 ± 3535.5^a^ | 197.5 ± 81.4 | 170.5 ± 94.9 |
|  | I | 0.0 ± 0.0 | 87.4 ± 65.6^a^ | 1474.2 ± 1159.7^a^ | 100.9 ± 36.6 | 634.6 ± 1419.0 | 3347.2 ± 3873.3^a^ | 99.8 ± 13.5^a^ | 98.2 ± 63.7^a^ |
|  | R | 0.0 ± 0.0 | 5681.7 ± 10372.4^b^ | 8196.4 ± 3841.0^b^ | 97.1 ± 10.2 | 304.4 ± 629.5 | 19118.0 ± 8398.9^b^ | 247.3 ± 140.1^b^ | 188.8 ± 65.6^b^ |
|  |  |  |  |  |  |  |  |  |  |
| PBMC  Day 66  (10 dpi 1 I;  10 dpi 2 R) | C | 0.0 ± 0.0 | 56.8 ± 9.4^a^ | 1397.5 ± 530.8^a^ | 170.0 ± 52.8 | 0.0 ± 0.0 | 2640.6 ± 1518.5 | 127.2 ± 30.7 | 95.3 ± 76.4 |
|  | I | 0.0 ± 0.0 | 49.4 ± 20.4^a^ | 741.0 ± 276.7^b^ | 145.6 ± 109.5 | 71.4 ± 159.7 | 1527.4 ± 875.4 | 135.6 ± 28.7 | 181.3 ± 112.3 |
|  | R | 30.8 ± 56.9 | 331.5 ± 245.6^b^ | 2518.0 ± 1581.9^a^ | 114.5 ± 45.2 | 9.0 ± 20.2 | 4695.8 ± 4504.7 | 161.3 ± 12.0 | 266.4 ± 272.0 |
|  |  |  |  |  |  |  |  |  |  |
| Spleen  Day 77  (21 dpi 1 I;  21 dpi 2 R) | C | 1585.6 ± 1033.0 | 7068.9 ± 11641.1 | 10898.8 ± 1108.4^a^ | 104.7 ± 64.8 | 841.3 ± 246.2^a^ | 41453.5 ± 11189.7 | 387.1 ± 127.7 | 3448.9 ± 2025.0 |
|  | I | 551.7 ± 542.9 | 805.9 ± 831.1^a^ | 3853.2 ± 2459.6^b^ | 83.5 ± 54.3 | 187.5 ± 195.5^b^ | 23832.8 ± 18335.3 | 221.5 ± 116.8 | 1394.1 ± 1012.0^a^ |
|  | R | 1905.7 ± 1797.5 | 9447.4 ± 14963.6^b^ | 7944.7 ± 1934.9^c^ | 80.7 ± 40.8 | 606.8 ± 405.2 | 32227.5 ± 12894.8 | 297.0 ± 148.0 | 4204.8 ± 2096.9^b^ |
|  |  |  |  |  |  |  |  |  |  |
| Cervical LN  Day 77  (21 dpi 1 I;  21 dpi 2 R) | C | 89.8 ± 179.7 | 50.0 ± 27.4 | 439.7 ± 322.0 | 81.6 ± 14.0 | 2.2 ± 4.4 | 2074.8 ± 1458.9 | 101.1 ± 28.8 | 291.1 ± 107.9^a^ |
|  | I | 21.5 ± 37.3 | 26.2 ± 6.7 | 117.5 ± 144.2 | 77.8 ± 17.6 | 0.0 ± 0.0 | 269.6 ± 112.2 | 73.3 ± 22.3 | 88.9 ± 124.0 |
|  | R | 0.0 ± 0.0 | 25.4 ± 11.3 | 35.1 ± 44.7 | 78.8 ± 28.2 | 0.0 ± 0.0 | 1001.4 ± 901.2 | 78.1 ± 34.2 | 55.4 ± 52.9^b^ |
|  |  |  |  |  |  |  |  |  |  |
| Pelvic LN  Day 77  (21 dpi 1 I;  21 dpi 2 R) | C | 0.0 ± 0.0 | 88.0 ± 45.8 | 343.1 ± 80.7 | 96.0 ± 45.0 | 0.0 ± 0.0 | 1431.0 ± 360.3 | 205.0 ± 77.8 | 746.0 ± 169.0 |
|  | I | 6.9 ± 15.4 | 87.0 ± 63.7 | 302.1 ± 331.0 | 94.6 ± 30.8 | 0.0 ± 0.0 | 1319.9 ± 1040.2 | 172.9 ± 54.4 | 621.8 ± 274.4 |
|  | R | 3.5 ± 7.8 | 94.2 ± 55.7 | 157.8 ± 180.8 | 56.1 ± 18.6 | 0.0 ± 0.0 | 847.1 ± 686.0 | 130.1 ± 47.4 | 515.9 ± 294.0 |

^a,b,c^ For each time point or tissue, means with a different superscript within a column are significantly different (*p* < 0.05).

* C: control group; I: infection group; R: re-infection group
