# Supplementary material for: The immune response against Chlamydia suis genital tract infection partially protects against re-infection
Source: Vet Res. 2014 Sep 25;45(1):95. doi: 10.1186/s13567-014-0095-6 (PMC4181727; doi:10.1186/s13567-014-0095-6)
Supplement: Additional file 5: — Mean cytokine concentration (pg/mL) ± standard deviation in culture medium of PBMC, isolated at 7 and 10 days post infection or re-infection, and of spleen, pelvic and cervical lymph node MC at euthanasia, measured at 72 h post stimulation with C. suis S45. Additional file 5 presents the results for cytokine detection at 72 h post stimulation. [file 13567_2014_95_MOESM5_ESM.docx]

|  | Group * | IFN-γ | TNF-α | IL-1β | IL-4 | IL-6 | IL-8 | IL-10 | IL-12p40 |
| --- | --- | --- | --- | --- | --- | --- | --- | --- | --- |
| PBMC  Day 7  (7 dpi 1 R) | C | 98.3 ± 99.2 | 627.8 ± 659.7^a^ | 4457.8 ± 1581.5^a^ | 139.5 ± 31.0^a^ | 611.6 ± 1039.7^a^ | 26444.8 ± 5082.7 | 757.9 ± 341.6 | 414.2 ± 159.7^a^ |
|  | I | 126.0 ± 157.3 | 855.3 ± 375.2^a^ | 7941.3 ± 3412.2 | 111.0 ± 6.5^b^ | 1336.7 ± 1309.2 | 22123.4 ± 5898.8 | 787.4 ± 325.6 | 392.2 ± 118.9^a^ |
|  | R | 2100.4 ± 1873.3 | 9288.1 ± 11716.4^b^ | 10353.4 ± 3454.7^b^ | 116.0 ± 45.3 | 3201.7 ± 2077.6^b^ | 30837.7 ± 14239.3 | 1138.9 ± 602.3 | 806.6 ± 192.4^b^ |
|  |  |  |  |  |  |  |  |  |  |
| PBMC  Day 10  (10 dpi 1 R) | C | 828.0 ± 1510.3 | 795.7 ± 1213.2 | 2980.8 ± 2345.0 | 67.5 ± 22.6 | 460.0 ± 920.0 | 14506.5 ± 11370.7 | 664.6 ± 434.3 | 368.2 ± 92.0 |
|  | I | 500.0 ± 518.1 | 683.2 ± 334.9 | 4761.5 ± 2852.7 | 109.9 ± 58.4 | 501.3 ± 554.3 | 15131.6 ± 2542.2 | 714.8 ± 203.7 | 383.5 ± 251.1 |
|  | R | 1713.0 ± 2204.0 | 961.6 ± 893.2 | 5100.2 ± 3593.4 | 109.1 ± 51.5 | 1689.8 ± 1641.5 | 20478.8 ± 8615.2 | 1112.6 ± 358.5 | 428.5 ± 154.9 |
|  |  |  |  |  |  |  |  |  |  |
| PBMC  Day 63  (7 dpi 1 I;  7 dpi 2 R) | C | 0.0 ± 0.0^a^ | 162.4 ± 198.0^a^ | 339.4 ± 256.6^a^ | 153.2 ± 51.5 | 64.9 ± 101.0^a^ | 11829.5 ± 10910.1 | 174.1 ± 55.7^a^ | 129.1 ± 69.9^a^ |
|  | I | 0.0 ± 0.0^a^ | 221.0 ± 137.6^a^ | 300.2 ± 322.0^a^ | 153.9 ± 55.1 | 192.8 ± 264.0^a^ | 7553.1 ± 5451.8 | 145.3 ± 66.4^a^ | 110.3 ± 9.5^a^ |
|  | R | 100.1 ± 80.3^b^ | 42189.2 ± 33858.1^b^ | 10025.3 ± 1628.5^b^ | 145.0 ± 81.5 | 2835.5 ± 988.4^b^ | 14127.9 ± 5192.9 | 547.2 ± 232.3^b^ | 300.2 ± 106.5^b^ |
|  |  |  |  |  |  |  |  |  |  |
| PBMC  Day 66  (10 dpi 1 I;  10 dpi 2 R) | C | 43.0 ± 86.1 | 1070.0 ± 1193.1^a^ | 1363.9 ± 956.9^a^ | 73.8 ± 20.6 | 1050.9 ± 1144.3^a^ | 20822.3 ± 14899.6 | 214.8 ± 104.0^a^ | 180.6 ± 75.7 |
|  | I | 72.9 ± 124.3 | 185.4 ± 74.2^a^ | 267.1 ± 168.8^b^ | 55.9 ± 12.6 | 0.2 ± 0.4^a^ | 8341.6 ± 6800.7^a^ | 82.6 ± 45.9^b^ | 90.1 ± 82.0^a^ |
|  | R | 2.8 ± 6.3 | 40175.6 ± 66573.5^b^ | 12831.1 ± 1533.1^c^ | 48.3 ± 15.8 | 5440.6 ± 1152.9^b^ | 20828.7 ± 5075.0^b^ | 390.7 ± 81.9^c^ | 340.4 ± 196.8^b^ |
|  |  |  |  |  |  |  |  |  |  |
| Spleen  Day 77  (21 dpi 1 I;  21 dpi 2 R) | C | 3924.4 ± 957.5^a^ | 7260.2 ± 8443.7^a^ | 3298.3 ± 630.8^a^ | 148.0 ± 81.7 | 2934.4 ± 842.2^a^ | 26199.6 ± 9525.2^a^ | 1086.8 ± 488.7 | 5458.1 ± 3593.8^a^ |
|  | I | 1560.1 ± 1373.9^b^ | 1061.1 ± 1055.8^b^ | 1018.9 ± 645.4^b^ | 67.5 ± 35.9 | 479.2 ± 315.1^b^ | 6762.8 ± 2120.6^b^ | 619.8 ± 447.6 | 2203.8 ± 1311.0^b^ |
|  | R | 5684.7 ± 2240.9^a^ | 3937.4 ± 3914.0^a^ | 2089.9 ± 872.9^a^ | 89.4 ± 36.3 | 2211.3 ± 961.1^a^ | 32102.3 ± 23143.1^a^ | 1035.2 ± 301.8 | 7853.9 ± 2388.3^a^ |
|  |  |  |  |  |  |  |  |  |  |
| Cervical LN  Day 77  (21 dpi 1 I;  21 dpi 2 R) | C | 660.9 ± 539.9 | 718.7 ± 493.4^a^ | 679.7 ± 479.5 | 97.2 ± 65.3 | 251.4 ± 196.6^a^ | 12858.2 ± 6186.6^a^ | 229.8 ± 77.2 | 1598.2 ± 860.2 |
|  | I | 186.8 ± 151.4 | 173.0 ± 148.2 | 119.7 ± 52.3 | 62.4 ± 57.5 | 0.0 ± 0.0^b^ | 1562.2 ± 562.7^b^ | 122.8 ± 32.4 | 627.0 ± 639.5 |
|  | R | 14.5 ± 25.0 | 62.5 ± 32.8^b^ | 98.60 ± 83.57 | 121.0 ± 18.2 | 0.0 ± 0.0^b^ | 5496.8 ± 8220.2 | 124.4 ± 52.1 | 660.5 ± 503.0 |
|  |  |  |  |  |  |  |  |  |  |
| Pelvic LN  Day 77  (21 dpi 1 I;  21 dpi 2 R) | C | 1191.9 ± 789.5 | 980.2 ± 405.6 | 506.6 ± 250.2 | 92.8 ± 21.4^a^ | 270.1 ± 291.2^a^ | 15994.2 ± 8742.3 | 446.5 ± 129.0 | 2098.6 ± 888.0 |
|  | I | 1070.2 ± 1239.9 | 706.0 ± 380.6 | 283.7 ± 149.0 | 60.4 ± 8.1^b^ | 5.2 ± 11.7^b^ | 3453.7 ± 2027.7 | 373.4 ± 286.1 | 1752.8 ± 1009.5 |
|  | R | 1193.4 ± 902.9 | 1007.1 ± 364.3 | 176.4 ± 163.6 | 103.1 ± 1.3 | 0.0 ± 0.0^b^ | 6748.2 ± 5004.3 | 406.9 ± 161.5 | 1698.4 ± 840.3 |

^a,b,c^ For each time point or tissue, means with a different superscript within a column are significantly different (*p* < 0.05).

* C: control group; I: infection group; R: re-infection group
